# Supplementary material for: Cholinergic modulation of hippocampal calcium activity across the sleep-wake cycle
Source: eLife. 2019 Mar 7;8:e39777. doi: 10.7554/eLife.39777 (PMC6435325; doi:10.7554/eLife.39777)
Supplement: Figure 1—source data 1. [file elife-39777-fig1-data1.docx]

**Figure 1-source data 1**

| **Figure 1I** | **Ca2+ rate (Hz)** | | | | |
| --- | --- | --- | --- | --- | --- |
|  | **Mouse** | **RUN** | **REST** | **SWS** | **REM** |
|  | 1 | 0.0320 | 0.0070 | 0.0065 | 0.0263 |
|  | 2 | 0.0358 | 0.0047 | 0.0036 | 0.0156 |
|  | 3 | 0.0313 | 0.0142 | 0.0074 | 0.0109 |
|  | 4 | 0.0222 | 0.0088 | 0.0013 | 0.0089 |
|  | 5 | 0.0148 | 0.0043 | 0.0048 | 0.0066 |
|  | | | | | |
| **Figure 1J** |  | **ΔF/F (Z-score)** | | | |
|  | **Mouse** | **RUN** | **REST** | **SWS** | **REM** |
|  | 1 | 3.7785 | 3.0433 | 2.8364 | 3.9186 |
|  | 2 | 3.1242 | 2.9645 | 2.7705 | 3.6990 |
|  | 3 | 3.7755 | 3.4505 | 3.2315 | 3.3089 |
|  | 4 | 3.9369 | 4.0353 | 3.4794 | 4.4616 |
|  | 5 | 3.4226 | 3.5309 | 3.4381 | 3.4439 |
|  | | | | | |
| **Figure 1L** |  | **Theta power** | | | |
|  | **Mouse** | **RUN** | **REST** | **SWS** | **REM** |
|  | 1 | 42118 | 20559 | 22585 | 43032 |
|  | 2 | 42653 | 28485 | 29060 | 47336 |
|  | 3 | 27382 | 10325 | 17368 | 50588 |
|  | 4 | 303620 | 114900 | 214760 | 393770 |
|  | 5 | 20763 | 6586 | 6695 | 22889 |
|  | | | | | |
| **Figure 1M** | **Fraction of Ca^2+^ bursts** | | | | |
|  | **Mouse** | **Still** | **Moving** |  | |
|  | 1 | 0.116 | 0.883 |  |  |
|  | 2 | 0.189 | 0.810 |  |  |
|  | 3 | 0.136 | 0.863 |  |  |
|  | 4 | 0.086 | 0.913 |  |  |
|  | 5 | 0.113 | 0.886 |  |  |
|  | 6 | 0.051 | 0.948 |  |  |
