# Supplementary material for: Cholinergic modulation of hippocampal calcium activity across the sleep-wake cycle
Source: eLife. 2019 Mar 7;8:e39777. doi: 10.7554/eLife.39777 (PMC6435325; doi:10.7554/eLife.39777)
Supplement: Figure 2—source data 1. [file elife-39777-fig2-data1.docx]

**Figure 2-source data 1**

| **Figure 2J** | **Ca2+ rate (Hz)-All events** | | | |
| --- | --- | --- | --- | --- |
|  | **Mouse** | **PRE** | **SWR** | **POST** |
|  | 1 | 0.0025 | 0.0029 | 0.0023 |
|  | 2 | 0.0021 | 0.0012 | 0.0021 |
|  | 3 | 0.0050 | 0.0019 | 0.0054 |
|  | 4 | 0.0063 | 0.0008 | 0.0049 |
|  | 5 | 0.0044 | 0.0027 | 0.0056 |
|  | 6 | 0.0044 | 0.0016 | 0.0045 |
|  | 7 | 0.0030 | 0.0012 | 0.0044 |
|  | 8 | 0.0045 | 0.0042 | 0.0056 |
|  | **Singlets** | | | |
|  | **Mouse** | **PRE** | **SWR** | **POST** |
|  | 1 | 0.0062 | 0.0038 | 0.0069 |
|  | 2 | 0.0049 | 0.0015 | 0.0052 |
|  | 3 | 0.0034 | 0 | 0.0046 |
|  | 4 | 0.0033 | 0.0013 | 0.0021 |
|  | 5 | 0.0022 | 0.0017 | 0.0027 |
|  | 6 | 0.0057 | 0.0021 | 0.0063 |
|  | 7 | 0.0043 | 0.0038 | 0.0047 |
|  | 8 | 0.0030 | 0.0047 | 0.0051 |
|  | **Trains** | | | |
|  | **Mouse** | **PRE** | **SWR** | **POST** |
|  | 1 | 0.0048 | 0.0035 | 0.0070 |
|  | 2 | 0.0042 | 0.0031 | 0.0064 |
|  | 3 | 0.0038 | 0.0062 | 0.0075 |
|  | 4 | 0.0021 | 0.0031 | 0.0032 |
|  | 5 | 0.0019 | 0 | 0.0023 |
|  | 6 | 0.0059 | 0.0052 | 0.0064 |
|  | 7 | 0.0038 | 0.0048 | 0.0073 |
|  | 8 | 0.0037 | 0 | 0.0073 |
|  | | | | |
| **Figure 2K** | **Neuropil (Z-score)** | | | |
|  | **Mouse** | **PRE** | **SWR** | **POST** |
|  | 1 | 0.0020 | 0.1774 | -0.1120 |
|  | 2 | 0.0011 | -0.0533 | -0.1875 |
|  | 3 | 0.0021 | 0.1784 | -0.2023 |
|  | 4 | 0.0258 | -0.0519 | -0.1710 |
|  | 5 | 0.0104 | 0.1142 | -0.0765 |
|  | 6 | -0.0007 | 0.0155 | -0.1071 |
|  | 7 | 0.0004 | 0.3099 | -0.1164 |
|  | **Singlets** | | | |
|  | **Mouse** | **PRE** | **SWR** | **POST** |
|  | 1 | -0.0081 | 0.1548 | 0.0169 |
|  | 2 | 0.0277 | 0.0467 | -0.0681 |
|  | 3 | 0.0434 | 0.0839 | -0.1225 |
|  | 4 | -0.0075 | 0.0542 | 0.0319 |
|  | 5 | -0.0090 | -0.0098 | -0.0651 |
|  | 6 | 0.0135 | -0.0971 | -0.1034 |
|  | 7 | 0.0068 | -0.0086 | 0.0576 |
|  | **Trains** | | | |
|  | **Mouse** | **PRE** | **SWR** | **POST** |
|  | 1 | -0.0021 | 0.1268 | -0.1829 |
|  | 2 | -0.0047 | 0.1230 | -0.0628 |
|  | 3 | -0.0084 | 0.2825 | -0.1722 |
|  | 4 | 0.0113 | 0.0028 | -0.0672 |
|  | 5 | 0.0348 | -0.3435 | -0.5267 |
|  | 6 | 0.0471 | -0.1888 | -0.1356 |
|  | 7 | -0.0198 | -0.1122 | -0.0154 |
