# Supplementary material for: Cholinergic modulation of hippocampal calcium activity across the sleep-wake cycle
Source: eLife. 2019 Mar 7;8:e39777. doi: 10.7554/eLife.39777 (PMC6435325; doi:10.7554/eLife.39777)
Supplement: Figure 2—figure supplement 3—source data 1. [file elife-39777-fig2-figsupp3-data1.docx]

**Figure 2-figure supplement 3-source data 1**

| **Latency (s)** | | |
| --- | --- | --- |
| **Mouse** | **fEPSP** | **Soma Calcium signal** |
| 1 | 0.0100 | 0.0490 |
| 2 | 0.0345 | 0.1000 |
| 3 | 0.0180 | 0.0492 |
