# Supplementary material for: Cholinergic modulation of hippocampal calcium activity across the sleep-wake cycle
Source: eLife. 2019 Mar 7;8:e39777. doi: 10.7554/eLife.39777 (PMC6435325; doi:10.7554/eLife.39777)
Supplement: Figure 3—source data 1. [file elife-39777-fig3-data1.docx]

**Figure 3-source data 1**

| **i.p. experiments** | | | | **i.h. experiments** | | | |
| --- | --- | --- | --- | --- | --- | --- | --- |
| **Figure 3J** | **Ca2+ rate (Hz)-i.p.** | | | **Figure 3T** | **Ca2+ rate (Hz)-i.h.** | | |
|  | **Mouse** | **Veh** | **CNO** |  |  |  |  |
|  | 1 | 0.0073 | 0.0147 |  | **Mouse** | **Veh** | **CNO** |
|  | 2 | 0.0048 | 0.0103 |  | 1 | 0.0159 | 0.0231 |
|  | 3 | 0.0109 | 0.0144 |  | 2 | 0.0146 | 0.0196 |
|  | 4 | 0.0093 | 0.0221 |  | 3 | 0.0302 | 0.0350 |
|  | 5 | 0.0114 | 0.0195 |  | 4 | 0.0236 | 0.0353 |
|  | 6 | 0.0136 | 0.0172 |  | 5 | 0.0087 | 0.0134 |
|  | | | | | | | |
| **Figure 3K** | **ΔF/F (Z-score)-i.p.** | | | **Figure 3U** | **ΔF/F (Z-score)-i.h.** | | |
|  | **Mouse** | **Veh** | **CNO** |  |  |  |  |
|  | 1 | 2.8557 | 3.1741 |  | **Mouse** | **Veh** | **CNO** |
|  | 2 | 2.6746 | 2.9076 |  | 1 | 3.3001 | 3.1453 |
|  | 3 | 3.7301 | 3.1603 |  | 2 | 3.4932 | 3.3986 |
|  | 4 | 2.9385 | 3.1564 |  | 3 | 3.2739 | 3.3303 |
|  | 5 | 3.4603 | 3.9310 |  | 4 | 3.2201 | 3.1747 |
|  | 6 | 3.9014 | 3.6591 |  | 5 | 3.1250 | 3.4780 |
|  | | | | | | | |
| **Figure 3L** | **HC MUA (Hz)- i.p.** | | | **Figure 3V** | **Velocity in Sleep Chamber (cm/s)** | | |
|  | **Mouse** | **Veh** | **CNO** |  | **Mouse** | **Veh** | **CNO** |
|  | 1 | 0 | 2.542 |  | 1 | 0.170 | 0.172 |
|  | 2 | 7.139 | 6.917 |  | 2 | 0.098 | 0.232 |
|  | 3 | 0 | 0.040 |  | 3 | 0.216 | 0.246 |
|  | 4 | 11.675 | 11.96 |  | 4 | 0.119 | 0.129 |
|  | 5 | 0.612 | 0.157 |  | | | |
|  | | | | | | | |
| **Figure 3M** | **Velocity in Sleep Chamber (cm/s)- i.p.** | | |  | | | |
|  | **Mouse** | **Veh** | **CNO** |  |  |  |  |
|  | 1 | 0.61 | 0.63 |  |  |  |  |
|  | 2 | 0.66 | 0.50 |  |  |  |  |
|  | 3 | 1.04 | 0.98 |  |  |  |  |
|  | 4 | 0.77 | 0.88 |  |  |  |  |
|  | | | | | | | |
| **Figure 3W** | **Ca2+ rates (Hz)-No hM3Dq-i.p.** | | | **Figure 3W** | **Ca^2+^ rates (Hz)-No hM3Dq-i.h.** | | |
|  | **Mouse** | **Veh** | **CNO** |  | **Mouse** | **Veh** | **CNO** |
|  | 1 | 0.0178 | 0.0159 |  | 1 | 0.0149 | 0.0154 |
|  | 2 | 0.0081 | 0.0108 |  | 2 | 0.0118 | 0.0111 |
|  | 3 | 0.0118 | 0.0104 |  | 3 | 0.0150 | 0.0135 |
|  | 4 | 0.0214 | 0.0209 |  | 4 | 0.0163 | 0.0206 |
|  | 5 | 0.0146 | 0.0125 |  | | | |
|  | | | | | | | |
| **Figure 3N** | **SWR Rate (Hz)- i.p.** | | |  | | | |
|  | **Mouse** | **Veh** | **CNO** |  |  |  |  |
|  | 1 | 0.322 | 0.242 |  |  |  |  |
|  | 2 | 0.395 | 0.076 |  |  |  |  |
|  | 3 | 0.258 | 0.135 |  |  |  |  |
|  | 4 | 0.185 | 0.094 |  |  |  |  |
|  | | | | | | | |
| **Figure 3O** | **Theta power (×10^3^)-i.p.** | | |  | | | |
|  | **Mouse** | **Veh** | **CNO** |  |  |  |  |
|  | 1 | 1.646 | 2.655 |  |  |  |  |
|  | 2 | 8.242 | 2.049 |  |  |  |  |
|  | 3 | 6.752 | 3.940 |  |  |  |  |
|  | 4 | 3.336 | 2.719 |  |  |  |  |
|  | 5 | 1.301 | 1.857 |  |  |  |  |
|  | | | | | | | |
| **Figure 3X** | **Ca^2+^ rates (Hz)-i.p.** | | |  | | | |
|  | **Mouse** | **CNO** | **CNO+Scop** |  |  |  |  |
|  | 1 | 0.0218 | 0.0074 |  |  |  |  |
|  | 2 | 0.0292 | 0.0133 |  |  |  |  |
|  | 3 | 0.0205 | 0.0155 |  |  |  |  |
|  | 4 | 0.0185 | 0.0115 |  |  |  |  |
|  | 5 | 0.0321 | 0.0292 |  |  |  |  |
|  | 6 | 0.0241 | 0.0114 |  |  |  |  |
|  | 7 | 0.0538 | 0.0211 |  |  |  |  |
|  | | | | | | | |
| **Figure 3Y** | **ΔF/F (Z-score)-i.p.** | | |  | | | |
|  | **Mouse** | **CNO** | **CNO+Scop** |  |  |  |  |
|  | 1 | 3.2440 | 2.7549 |  |  |  |  |
|  | 2 | 3.0979 | 2.5832 |  |  |  |  |
|  | 3 | 3.1823 | 2.8864 |  |  |  |  |
|  | 4 | 3.3132 | 2.9756 |  |  |  |  |
|  | 5 | 3.8065 | 3.2279 |  |  |  |  |
