# Supplementary material for: Cholinergic modulation of hippocampal calcium activity across the sleep-wake cycle
Source: eLife. 2019 Mar 7;8:e39777. doi: 10.7554/eLife.39777 (PMC6435325; doi:10.7554/eLife.39777)
Supplement: Figure 3—figure supplement 2—source data 1. [file elife-39777-fig3-figsupp2-data1.docx]

**Figure 3-figure supplement 2-source data 1**

| **Time moving (s)-i.p.** | | |
| --- | --- | --- |
| **Mouse** | **Veh** | **CNO** |
| 1 | 294 | 183 |
| 2 | 400 | 724 |
| 3 | 516 | 365 |
| 4 | 660 | 406 |
| **Time still (s)-i.p.** | | |
| **Mouse** | **Veh** | **CNO** |
| 1 | 1050 | 1305 |
| 2 | 330 | 505 |
| 3 | 624 | 630 |
| 4 | 155 | 450 |
| **Time exploring (s)-i.p.** | | |
| **Mouse** | **Veh** | **CNO** |
| 1 | 62 | 10 |
| 2 | 280 | 250 |
| 3 | 75 | 150 |
| 4 | 450 | 300 |
| **Time grooming (s)-i.p.** | | |
| **Mouse** | **Veh** | **CNO** |
| 1 | 17 | 75 |
| 2 | 70 | 8 |
| 3 | 8 | 31 |
| 4 | 87 | 156 |
