# Supplementary material for: Cholinergic modulation of hippocampal calcium activity across the sleep-wake cycle
Source: eLife. 2019 Mar 7;8:e39777. doi: 10.7554/eLife.39777 (PMC6435325; doi:10.7554/eLife.39777)
Supplement: Figure 3—figure supplement 3—source data 1. [file elife-39777-fig3-figsupp3-data1.docx]

**Figure 3-figure supplement 3-source data 1**

| **Velocity in track (cm/s)-i.p.** | | |
| --- | --- | --- |
| **Mouse** | **Veh** | **CNO** |
| 1 | 10.51 | 10.74 |
| 2 | 10.59 | 11.45 |
| 3 | 13.52 | 12.06 |
| 4 | 10.40 | 12.79 |
| **% time in run-i.p.** | | |
| **Mouse** | **Veh** | **CNO** |
| 1 | 33.62 | 31.25 |
| 2 | 39.69 | 27.12 |
| 3 | 65.95 | 61.70 |
| 4 | 53.56 | 53.22 |
