# Supplementary material for: Cholinergic modulation of hippocampal calcium activity across the sleep-wake cycle
Source: eLife. 2019 Mar 7;8:e39777. doi: 10.7554/eLife.39777 (PMC6435325; doi:10.7554/eLife.39777)
Supplement: Figure 3—figure supplement 4—source data 1. [file elife-39777-fig3-figsupp4-data1.docx]

**Figure 3-figure supplement 4-source data 1**

| **ΔF/F (Z-score)-No hM3Dq-i.p.** | | |
| --- | --- | --- |
| **Mouse** | **Veh** | **CNO** |
| 1 | 3.7236 | 3.7154 |
| 2 | 3.4494 | 3.4525 |
| 3 | 3.7189 | 3.8657 |
| 4 | 4.5913 | 4.4908 |
| 5 | 3.2810 | 3.1404 |
| **ΔF/F (Z-score)-No hM3Dq-i.h.** | | |
| **Mouse** | **Veh** | **CNO** |
| 1 | 3.2393 | 3.2546 |
| 2 | 4.4133 | 3.7711 |
| 3 | 4.2988 | 3.9710 |
| 4 | 3.8892 | 3.6926 |
