# Supplementary material for: Cholinergic modulation of hippocampal calcium activity across the sleep-wake cycle
Source: eLife. 2019 Mar 7;8:e39777. doi: 10.7554/eLife.39777 (PMC6435325; doi:10.7554/eLife.39777)
Supplement: Figure 4—source data 1. [file elife-39777-fig4-data1.docx]

**Figure 4-source data 1**

| **i.p. experiments** | | | | **i.h. experiments** | | | |
| --- | --- | --- | --- | --- | --- | --- | --- |
| **Figure 4F** | **Ca2+ rate (Hz)-i.p.** | | | **Figure 4Q** | **Ca2+ rate (Hz)-i.h.** | | |
|  | **Mouse** | **Veh** | **Scopolamine** |  | **Mouse** | **Veh** | **Scopolamine** |
|  | 1 | 0.0218 | 0.0074 |  | 1 | 0.0175 | 0.0146 |
|  | 2 | 0.0292 | 0.0133 |  | 2 | 0.0189 | 0.0127 |
|  | 3 | 0.0205 | 0.0155 |  | 3 | 0.0287 | 0.0151 |
|  | 4 | 0.0185 | 0.0115 |  | 4 | 0.0307 | 0.0096 |
|  | 5 | 0.0321 | 0.0292 |  | 5 | 0.0144 | 0.0092 |
|  | 6 | 0.0241 | 0.0114 |  | | | |
|  | 7 | 0.0538 | 0.0211 |  |  |  |  |
|  | | | | | | | |
| **Figure 4G** | **ΔF/F (Z-score)-i.p.** | | | **Figure 4R** | **ΔF/F (Z-score)-i.h.** | | |
|  | **Mouse** | **Veh** | **Scopolamine** |  | **Mouse** | **Veh** | **Scopolamine** |
|  | 1 | 3.9778 | 3.2090 |  | 1 | 3.9170 | 3.1883 |
|  | 2 | 3.0923 | 2.6550 |  | 2 | 3.3102 | 3.0307 |
|  | 3 | 3.2572 | 2.9276 |  | 3 | 4.4776 | 3.2734 |
|  | 4 | 3.3818 | 2.8464 |  | 4 | 3.1096 | 3.0549 |
|  | 5 | 3.2411 | 2.8685 |  | 5 | 3.8622 | 2.9991 |
|  | 6 | 3.3138 | 2.7641 |  | | | |
|  | 7 | 3.7435 | 2.9232 |  |  |  |  |
|  | | | | | | | |
| **Figure 4H** | **HC MUA (Hz)-i.p.-Run** | | | **Figure 4S** | **Velocity in sleep chamber (cm/s)-i.h.** | | |
|  | **Mouse** | **Veh** | **Scopolamine** |  | **Mouse** | **Veh** | **Scopolamine** |
|  | 1 | 2.636 | 3.819 |  | 1 | 0.14 | 0.10 |
|  | 2 | 7.299 | 9.868 |  | 2 | 0.95 | 0.75 |
|  | 3 | 6.531 | 5.248 |  | 3 | 0.19 | 0.06 |
|  | 4 | 6.914 | 7.619 |  | 4 | 0.14 | 0.13 |
|  | 5 | 0.065 | 2.195 |  | 5 | 0.21 | 0.18 |
|  | 6 | 3.666 | 5.611 |  | | | |
|  | | | | | | | |
| **Figure 4I** | **SWR Rate (Hz)** | | |  | | | |
|  | **Mouse** | **Veh** | **Scopolamine** |  |  |  |  |
|  | 1 | 0.279 | 0.768 |  |  |  |  |
|  | 2 | 0.923 | 1.411 |  |  |  |  |
|  | 3 | 0.010 | 0.044 |  |  |  |  |
|  | 4 | 1.081 | 1.512 |  |  |  |  |
|  | 5 | 0.342 | 0.446 |  |  |  |  |
|  | 6 | 0.388 | 0.733 |  |  |  |  |
|  | | | | | | | |
| **Figure 4J** | **Velocity in run (cm/s)** | | |  | | | |
|  | **Mouse** | **Veh** | **Scopolamine** |  |  |  |  |
|  | 1 | 12.24 | 11.06 |  |  |  |  |
|  | 2 | 12.00 | 10.51 |  |  |  |  |
|  | 3 | 13.83 | 10.56 |  |  |  |  |
|  | 4 | 10.08 | 11.01 |  |  |  |  |
|  | 5 | 8.92 | 7.18 |  |  |  |  |
|  | 6 | 9.40 | 8.16 |  |  |  |  |
|  | 7 | 14.39 | 12.15 |  |  |  |  |
|  | | | | | | | |
| **Figure 4K** | **% time in run** | | |  | | | |
|  | **Mouse** | **Veh** | **Scopolamine** |  |  |  |  |
|  | 1 | 72.4 | 71.4 |  |  |  |  |
|  | 2 | 68.1 | 49.9 |  |  |  |  |
|  | 3 | 68.0 | 51.6 |  |  |  |  |
|  | 4 | 64.9 | 76.6 |  |  |  |  |
|  | 5 | 37.3 | 35.2 |  |  |  |  |
|  | 6 | 37.7 | 62.2 |  |  |  |  |
|  | 7 | 32.4 | 33.7 |  |  |  |  |
