# Supplementary material for: Cholinergic modulation of hippocampal calcium activity across the sleep-wake cycle
Source: eLife. 2019 Mar 7;8:e39777. doi: 10.7554/eLife.39777 (PMC6435325; doi:10.7554/eLife.39777)
Supplement: Figure 4—figure supplement 1—source data 1. [file elife-39777-fig4-figsupp1-data1.docx]

**Figure 4-figure supplement 1-source data 1**

| **Time moving (s)-i.p.** | | |
| --- | --- | --- |
| **Mouse** | **Veh** | **Scopolamine** |
| 1 | 363 | 594 |
| 2 | 384 | 152 |
| 3 | 224 | 72 |
| 4 | 375 | 378 |
| **Time still (s)-i.p.** | | |
| **Mouse** | **Veh** | **Scopolamine** |
| 1 | 1368 | 838 |
| 2 | 230 | 205 |
| 3 | 220 | 850 |
| 4 | 36 | 30 |
|  | | |
| **Time exploring (s)-i.p.** | | |
| **Mouse** | **Veh** | **Scopolamine** |
| 1 | 205 | 385 |
| 2 | 770 | 555 |
| 3 | 150 | 15 |
| 4 | 435 | 890 |
| **Time grooming (s)-i.p.** | | |
| **Mouse** | **Veh** | **Scopolamine** |
| 1 | 20 | 0 |
| 2 | 18 | 36 |
| 3 | 30 | 77 |
| 4 | 13 | 0 |
