# Supplementary material for: Cholinergic modulation of hippocampal calcium activity across the sleep-wake cycle
Source: eLife. 2019 Mar 7;8:e39777. doi: 10.7554/eLife.39777 (PMC6435325; doi:10.7554/eLife.39777)
Supplement: Figure 4—figure supplement 2—source data 1. [file elife-39777-fig4-figsupp2-data1.docx]

**Figure 4-figure supplement 2-source data 1**

| **Velocity in Sleep Chamber (cm/s)-i.p.** | | |
| --- | --- | --- |
| **Mouse** | **Veh** | **Scopolamine** |
| 1 | 0.61 | 0.58 |
| 2 | 1.01 | 0.79 |
| 3 | 1.06 | 0.90 |
| 4 | 0.96 | 1.07 |
| 5 | 0.62 | 0.52 |
| 6 | 0.57 | 0.76 |
| 7 | 0.61 | 0.71 |
