# Supplementary material for: Cholinergic modulation of hippocampal calcium activity across the sleep-wake cycle
Source: eLife. 2019 Mar 7;8:e39777. doi: 10.7554/eLife.39777 (PMC6435325; doi:10.7554/eLife.39777)
Supplement: Supplementary file 1. [file elife-39777-supp1.docx]

**Statistical Analyses for Group Data**

|  | **Bar** | **Size**  **(n)** | **Comparison** | **Analyses** | **T or F value** | ***P* value** |
| --- | --- | --- | --- | --- | --- | --- |
| **Figure 1** | | | | | | |
| Two animals with linear (LIN) probe and three animals with stereotrodes (ST) | | | | | | |
| **I** | Calcium rate (Hz) | | | Repeated ANOVA^#^ | F(3,12)=17.97 | <0.001*** |
|  | RUN | 5 | RUN *vs.*REST | *post hoc* |  | = 0.007** |
|  | REST |  | RUN *vs.* SWS | *post hoc* |  | = 0.003** |
|  | SWS |  | RUN *vs.* REM | *post hoc* |  | = 0.011* |
|  | REM |  | REST *vs*. SWS | *post hoc* |  | = 0.142 |
|  |  |  | REST *vs.*REM | *post hoc* |  | = 0.225 |
|  |  |  | SWS *vs.* REM | *post hoc* |  | = 0.050 |
| **J** | Calcium amplitude ΔF/F (Z-score) | | | Repeated ANOVA^#^ | F(3,12)=4.488 | = 0.025* |
|  | RUN | 5 | RUN *vs.*REST | *post hoc* |  | = 0.264 |
|  | REST |  | RUN *vs.* SWS | *post hoc* |  | = 0.042* |
|  | SWS |  | RUN *vs.* REM | *post hoc* |  | = 0.449 |
|  | REM |  | REST *vs*. SWS | *post hoc* |  | = 0.032* |
|  |  |  | REST *vs.*REM | *post hoc* |  | = 0.157 |
|  |  |  | SWS *vs.* REM | *post hoc* |  | = 0.059 |
| **K** | HC MUA (Hz) | | | Repeated ANOVA^#^ | F(2,6)=0.591 | =0.544 |
|  | REST | 4 | REST *vs*. SWS |  |  |  |
|  | SWS |  | REST *vs.*REM |  |  |  |
|  | REM |  | SWS *vs.* REM |  |  |  |
| **L** | Log (theta power) | | | Repeated ANOVA^#^ | F(2,8)=20.468 | =0.001** |
|  | RUN | 5 | RUN *vs.* REST | *post hoc* |  | =0.003** |
|  | REST |  | RUN *vs.* SWS | *post hoc* |  | =0.015* |
|  | SWS |  | REST *vs.* SWS. | *post hoc* |  | =0.124 |
|  | REM | Not used in ANOVA because we define REM phase in artificial manner. | | | | |
| **M** | Fraction of calcium bursts across distinct behavioural states | | |  |  |  |
|  | Still | 6 | Still *vs.*moving | Paired t-test | T=20.178 | <0.001*** |
|  | Moving |  |  |  |  |  |
| **Figure 2** | | | | | | |
| Four animals with LIN probe and four animals with ST | | | | | | |
| **J** | Calcium rate (Hz)-All events | | | Repeated ANOVA^#^ | F(2,14)=10.802 | =0.004** |
|  | Pre | 8 | Pre *vs.* SWR | *post hoc* |  | =0.022* |
|  | SWR |  | Pre *vs.* Post | *post hoc* |  | =0.292 |
|  | Post |  | Post *vs.* SWR | *post hoc* |  | =0.005** |
|  | Calcium rate (Hz)-Singlets | | | Repeated ANOVA^#^ | F(2,14)=9.628 | =0.005** |
|  | Pre | 8 | Pre *vs.* SWR | *post hoc* |  | =0.033* |
|  | SWR |  | Pre *vs.* Post | *post hoc* |  | =0.105 |
|  | Post |  | Post *vs.* SWR | *post hoc* |  | =0.007** |
|  | Calcium rate (Hz)-Trains | | |  | F(2,14)=9.145 | =0.003** |
|  | Pre | 8 | Pre *vs.* SWR |  |  | =0.447 |
|  | SWR |  | Pre *vs.* Post |  |  | =0.003** |
|  | Post |  | Post *vs.* SWR |  |  | =0.010* |
| **K** | Neuropil ΔF/F (Z-score)-All events | | | Repeated ANOVA^#^ | F(2,12)=15.627 | =0.003** |
|  | Pre | 7 | Pre *vs.* SWR | *post hoc* |  | =0.130 |
|  | SWR |  | Pre *vs.* Post | *post hoc* |  | <0.001*** |
|  | Post |  | Post *vs.* SWR | *post hoc* |  | =0.003** |
|  | Neuropil ΔF/F (Z-score)-Singlets | | | Repeated ANOVA^#^ | F(2,12)=2.242 | =0.149 |
|  | Pre | 7 | Pre *vs.* SWR | *post hoc* |  |  |
|  | SWR |  | Pre *vs.* Post | *post hoc* |  |  |
|  | Post |  | Post *vs.* SWR | *post hoc* |  |  |
|  | Neuropil ΔF/F (Z-score)-Trains | | | Repeated ANOVA^#^ | F(2,12)=2.984 | =0.089 |
|  | Pre | 7 | Pre *vs.* SWR | *post hoc* |  |  |
|  | SWR |  | Pre *vs.* Post | *post hoc* |  |  |
|  | Post |  | Post *vs.* SWR | *post hoc* |  |  |
| **Figure 3** | | | | | | |
| Five animals with LIN probe | | | | | | |
| **J** | Calcium rate | | |  |  |  |
|  | Veh-i.p. | 6 | Veh *vs.* CNO | Paired t-test | T=4.747 | =0.005** |
|  | CNO-i.p. |  |  |  |  |  |
| **K** | Calcium amplitude (ΔF/F) | | |  |  |  |
|  | Veh-i.p. | 6 | Veh *vs.* CNO | Paired t-test | T=0.442 | =0.677 |
|  | CNO-i.p. |  |  |  |  |  |
| **L** | HC MUA (Hz) | | |  |  |  |
|  | Veh-i.p. | 5 | Veh *vs.* CNO | Paired t-test | T=0.814 | =0.461 |
|  | CNO-i.p. |  |  |  |  |  |
| **M** | Velocity in sleep chamber (cm/s) | | |  |  |  |
|  | Veh-i.p. | 7 | Veh *vs.* CNO | Paired t-test | T=0.329 | =0.753 |
|  | CNO-i.p. |  |  |  |  |  |
| **N** | SWR rate (Hz) | | |  |  |  |
|  | Veh-i.p. | 4 | Veh *vs.* CNO | Paired t-test^&^ | T=3.695 | =0.035* |
|  | CNO-i.p. |  |  |  |  |  |
| **O** | Theta power | | |  |  |  |
|  | Veh-i.p. | 5 | Veh *vs.* CNO | Paired t-test | T=1.218 | =0.290 |
|  | CNO-i.p. |  |  |  |  |  |
| **T** | Calcium rate (Hz) | | |  |  |  |
|  | Veh-i.h. | 5 | Veh *vs.* CNO | Paired t-test | T=4.972 | =0.008** |
|  | CNO-i.h. |  |  |  |  |  |
| **U** | Calcium amplitude (ΔF/F) | | |  |  |  |
|  | Veh-i.h. | 5 | Veh *vs.* CNO | Paired t-test | T=0.256 | =0.810 |
|  | CNO-i.h. |  |  |  |  |  |
| **V** | Velocity in sleep chamber (cm/s) | | |  |  |  |
|  | Veh-i.h. | 4 | Veh *vs.* CNO | Paired t-test | T=1.431 | =0.248 |
|  | CNO-i.h. |  |  |  |  |  |
| **W** | Calcium rate (Hz)- No hM3Dq | | |  |  |  |
|  | Veh-i.p. | 5 | Veh *vs.* CNO | Paired t-test | T=0.747 | =0.496 |
|  | CNO-i.p. |  |  |  |  |  |
|  | Veh-i.h. | 4 | Veh *vs.* CNO | Paired t-test | T=0.528 | =0.637 |
|  | CNO-i.h. |  |  |  |  |  |
| **X** | Calcium rate (Hz) | | |  |  |  |
|  | CNO | 7 | CNO *vs.* CNO+Scop | Paired t-test | T=3.428 | =0.014* |
|  | CNO+Scop |  |  |  |  |  |
| **Y** | Calcium amplitude (ΔF/F) | | |  |  |  |
|  | CNO | 5 | CNO *vs.* CNO+Scop | Paired t-Test | T=8.201 | =0.001** |
|  | CNO+Scop |  |  |  |  |  |
| **Figure 3-figure supplement 2** | | | | | | |
| **A** | Time moving (% Veh) | | |  |  |  |
|  | Veh-i.p. | 4 | Veh *vs.* CNO | Paired t-Test | T=0.376 | =0.732 |
|  | CNO-i.p. |  |  |  |  |  |
| **B** | Time still (% Veh) | | |  |  |  |
|  | Veh-i.p. | 4 | Veh *vs.* CNO | Paired t-Test | T=2.856 | =0.065 |
|  | CNO-i.p. |  |  |  |  |  |
| **C** | Time exploring (% Veh) | | |  |  |  |
|  | Veh-i.p. | 4 | Veh *vs.* CNO | Paired t-Test | T=0.936 | =0.418 |
|  | CNO-i.p. |  |  |  |  |  |
| **D** | Time grooming (% Veh) | | |  |  |  |
|  | Veh-i.p. | 4 | Veh *vs.* CNO | Paired t-Test | T=0.742 | =0.512 |
|  | CNO-i.p |  |  |  |  |  |
| **Figure 3-figure supplement 3** | | | | | | |
| **A** | Run velocity in track (cm/s) | | |  |  |  |
|  | Veh-i.p. | 4 | Veh *vs.* CNO | Paired t-Test | T=0.632 | =0.572 |
|  | CNO-i.p. |  |  |  |  |  |
| **B** | % Time in run | | |  |  |  |
|  | Veh-i.p. | 4 | Veh *vs.* CNO | Paired t-Test | T=1.819 | =0.167 |
|  | CNO-i.p. |  |  |  |  |  |
| **Figure 3-figure supplement 4** | | | | | | |
| **A** | Calcium amplitude (ΔF/F) | | |  |  |  |
|  | Veh-i.p. | 5 | Veh *vs.* CNO | Paired t-Test | T=0.400 | =0.710 |
|  | CNO-i.p. |  |  |  |  |  |
| **B** | Calcium amplitude (ΔF/F) | | |  |  |  |
|  | Veh-i.h. | 4 | Veh *vs.* CNO | Paired t-Test | T=2.091 | =0.128 |
|  | CNO-i.h. |  |  |  |  |  |
| **Figure 4** | | | | | | |
| Five animals with LIN probe and one animal with ST | | | | | | |
| **F** | Calcium rate (Hz) | | |  |  |  |
|  | Veh-i.p. | 7 | Veh *vs.* Scop | Paired t-test | T=3.428 | =0.014* |
|  | Scop-i.p. |  |  |  |  |  |
| **G** | Calcium amplitude (ΔF/F) | | |  |  |  |
|  | Veh -i.p. | 7 | Veh *vs.* Scop | Paired t-test | T=7.638 | <0.001*** |
|  | Scop-i.p. |  |  |  |  |  |
| **H** | HC MUA (Hz)-Run | | |  |  |  |
|  | Veh -i.p. | 6 | Veh *vs.* Scop | Paired t-test | T=2.124 | =0.087 |
|  | Scop-i.p. |  |  |  |  |  |
| **I** | SWR rate (Hz) | | |  |  |  |
|  | Veh -i.p. | 6 | Veh *vs.* Scop | Paired t-test | T=3.879 | =0.012* |
|  | Scop-i.p. |  |  |  |  |  |
| **J** | Run velocity in track (cm/s) | | |  |  |  |
|  | Veh -i.p. | 7 | Veh *vs.* Scop | Paired t-test | T=3.036 | =0.023* |
|  | Scop-i.p. |  |  |  |  |  |
| **K** | % Time in run | | |  |  |  |
|  | Veh -i.p. | 7 | Veh *vs.* Scop | Paired t-test | T=0.0 | =1.0 |
|  | Scop-i.p. |  |  |  |  |  |
| **L** | Log(Theta power) | | |  |  |  |
|  | Veh -i.p. | 7 | Veh *vs.* Scop | Paired t-test | T=2.443 | =0.050 |
|  | Scop-i.p. |  |  |  |  |  |
| **Q** | Calcium rate (Hz) | | |  |  |  |
|  | Veh -i.h. | 5 | Veh *vs.* Scop | Paired t-test | T=2.923 | =0.043* |
|  | Scop-i.h. |  |  |  |  |  |
| **R** | Calcium amplitude (ΔF/F) | | |  |  |  |
|  | Veh -i.h. | 5 | Veh *vs.* Scop | Paired t-test | T=3.041 | =0.038* |
|  | Scop-i.h. |  |  |  |  |  |
| **S** | Velocity in sleeping box (cm/s) | | |  |  |  |
|  | Veh -i.h. | 5 | Veh *vs.* Scop | Paired t-test | T=2.318 | =0.081 |
|  | Scop-i.h. |  |  |  |  |  |
| **Figure 4-figure supplement 1** | | | | | | |
| **A** | Time moving (% Veh) | | |  |  |  |
|  | Veh-i.p. | 4 | Veh *vs.* Scop | Paired t-Test | T=0.529 | =0.634 |
|  | Scop-i.p. |  |  |  |  |  |
| **B** | Time still (% Veh) | | |  |  |  |
|  | Veh-i.p. | 4 | Veh *vs.* Scop | Paired t-Test | T=0.073 | =0.947 |
|  | Scop-i.p. |  |  |  |  |  |
| **C** | Time exploring (% Veh) | | |  |  |  |
|  | Veh-i.p. | 4 | Veh *vs.* Scop | Paired t-Test | T=1.109 | =0.348 |
|  | Scop-i.p. |  |  |  |  |  |
| **D** | Time grooming (% Veh) | | |  |  |  |
|  | Veh-i.p. | 4 | Veh *vs.* Scop | Paired t-Test | T=0.519 | 0.639 |
|  | Scop-i.p. |  |  |  |  |  |
| **Figure 4-figure supplement 2** | | | | | | |
|  | Velocity in sleeping box (cm/s) | | |  |  |  |
|  | Veh-i.p. | 4 | Veh *vs.* Scop | Paired t-Test | T=0.311 | =0.766 |
|  | Scop-i.p. |  |  |  |  |  |
| **Other:** Mentioned data not in figures | | | | | | |
|  | Velocity during SWR events (cm/s) | | | Repeated ANOVA^#^ | F(2,12)=1.71 | =0.23 |
|  | Pre | 7 | Pre *vs.* SWR |  |  |  |
|  | SWR |  | Pre *vs.* Post |  |  |  |
|  | Post |  | Post *vs.* SWR |  |  |  |
|  | HC MUA (Hz)-Rest | | |  |  |  |
|  | Veh -i.p. | 6 | Veh *vs.* Scop | Paired t-test | T=0.177 | =0.866 |
|  | Scop-i.p. |  |  |  |  |  |
|  | Calcium rate in rest state | | |  |  |  |
|  | Veh1 | 3 | Veh1 *vs*.Veh2 | Paired t-test | T=0.550 | =0.638 |
|  | Veh2 |  |  |  |  |  |
|  | Calcium rate in run state | | |  |  |  |
|  | Veh1 | 3 | Veh1 *vs.*Veh2 | Paired t-test | T=1.599 | =0.251 |
|  | Veh2 |  |  |  |  |  |
|  | Calcium amplitude (ΔF/F) in rest state | | |  |  |  |
|  | Veh1 | 3 | Veh1 *vs.*Veh2 | Paired t-test | T=2.935 | =0.099 |
|  | Veh2 |  |  |  |  |  |
|  | Calcium amplitude (ΔF/F) in run state | | |  |  |  |
|  | Veh1 | 3 | Veh1 *vs.*Veh2 | Paired t-test | T=2.793 | =0.108 |
|  | Veh2 |  |  |  |  |  |

^#^ Huynh-Feldt adjustment, LIN = Linear probe, ST = Stereotrode. *P<0.05, **P<0.01 and ***P<0.001. ^&^one tailed paired t-test
